# A Scoping Review of Alternative Payment Models in Maternity Care: Insights in Key Design Elements and Effects on Health and Spending

**DOI:** 10.5334/ijic.5535

**Published:** 2021-04-21

**Authors:** Eline F. de Vries, Zoë T.M. Scheefhals, Mieneke de Bruin-Kooistra, Caroline A. Baan, Jeroen N. Struijs

**Affiliations:** 1 Tranzo, Tilburg School of Social and Behavioral Sciences, Tilburg University; 2 Department of Quality of Care and Health Economics, Center of Prevention, Nutrition and Health Services Research, National Institute for Public Health and the Environment; Mailing address: PO Box 1, 3720 BA Bilthoven, the Netherlands; 3 Department for Public Health and Primary Care, LUMC Campus The Hague, Leiden University Medical Center; 4 Ministry of Health, Welfare and Sport; the Netherlands

**Keywords:** alternative payment models, value-based health care, maternity care, APM, value-based payment, payment reform, review

## Abstract

**Introduction:**

Although effects of alternative payment models on health outcomes and health spending are unclear, they are increasingly implemented in maternity care. We aimed to provide an overview of alternative payment models implemented in maternity care, describing their key design elements among which the type of APM, the care providers that participate in the model, populations and care services that are included and the applied risk mitigation strategies. Next to that, we made an inventory of the empirical evidence on the effects of APMs on maternal and neonatal health outcomes and spending on maternity care.

**Methods:**

We searched PubMed, Embase and Scopus databases for articles published from January 2007 through October 2020. Search key words included ‘alternative payment model’, ‘value based payment model’, ‘obstetric’, ‘maternity’. English or Dutch language articles were included if they described or empirically evaluated initiatives implementing alternative payment models in maternity care in high-income countries. Additional relevant documents were identified through reference tracking. We systematically analyzed the initiatives found and examined the evidence regarding health outcomes and health spending. The process was guided by the Preferred Reporting Items for Systematic Reviews and Meta-Analysis (PRISMA) to ensure validity and reliability.

**Results:**

We identified 17 initiatives that implemented alternative payment models in maternity care. Thirteen in the United States, two in the United Kingdom, one in New Zealand and one in the Netherlands. Within these initiatives three types of alternative payment models were implemented; pay-for-performance (n = 2), shared savings models (n = 7) and bundled payment models (n = 8). Alternative payment models that shifted more financial accountability towards providers seemed to include more strategies that mitigated those risks. Risk mitigation strategies were applied to the included population, included services or at the level of total expenditures. Of these seventeen initiatives, we found four empirical effect studies published in peer-reviewed journals. Three of them were of moderate quality and one weak. Two studies described an association of the alternative payment model with an improvement of specific health outcomes and two studies described a reduction in medical spending.

**Conclusions:**

This study shows that key design elements of alternative payment models including risk mitigation strategies vary highly. Risk mitigation strategies seem to be relevant tools to increase APM uptake and protect providers from (initially) bearing too much (perceived) financial risk. Empirical evidence on the effects of APMs on health outcomes and spending is still limited. A clear definition of key design elements and a further, in-depth, understanding of key design elements and how they operate into different health settings is required to shape payment reform that aligns with its goals.

## Introduction

Policies around the world aim for the reduction of avoidable infant mortality, pre-term rates and maternal mortality. Previous reports show that improvements can be made by optimizing the delivery of health services [1,2]. For instance, low-value services, such as non-medically indicated caesarean sections, are increasingly performed, while high-value services, such as screening for gestational diabetes or educating women on what to expect during and after birth, are underutilized [2,3]. To achieve optimally organized care, more coordination of care delivery is needed.

The promise of alternative payment models (APMs) is that they incentivize care coordination, and stimulate the use of high-value care and discourage the use of low-value care by increased provider accountability. In the literature, theoretical effects of APMs have been discussed [4,5,6,7,8] in comparison to effects of fee-for-service (FFS) models, which are commonly used in daily practice. The financial risks in the FFS models are borne largely by the payers. Since health care providers thereby run no (or marginal) financial risk in terms of the volume and the value of care they deliver, a FFS system inadvertently encourages providers to deliver larger volumes of care and low-value care [4,5,6,7,9]. APMs aim to remove these incentives by shifting the accountability, for both health outcomes and health spending, towards providers [4]. This shift towards providers serves as an incentive to avoid unnecessary care as well as encourages other cost-conscious behaviors such as downward substitution of care, task reallocation and more efficient coordination between practitioners within care. The scope of the APM determines the allocation of financial risk between provider and payer. The scope is defined both by the type of APM and by custom design features such as risk mitigation strategies that can be applied. Ideally, the performance risk (i.e. risks that are related to the providers own share in providing high quality and efficient care [7]) is allocated with the provider and the insurance risk (i.e. risks that stem from patients and their respective needs [7]) with the payer. Performance risk increases the incentives to create value, whereas insurance risk increases a providers’ level of financial risk without the provider being able to control it. Therefore, the optimal allocation of risks within an APM is where, for providers, insurance risk is minimized and the performance risk is maximized [8].

Based on these theoretical advantages of APMs, both payers and providers are generally willing to adopt APMs [10,11]. However, the number of APMs that have been implemented is currently still low and there is no strong empirical evidence that supports the theory. Possibly, the (perceived) risk and uncertainty for providers is too high and restraints them from adopting APMs [10]. Strategies that lower the (perceived) risk for providers may help to stimulate APM adoption. Such risk mitigation strategies are for example stop loss provisions (e.g. the exclusion of high-risk patients or high-cost services) or adding risk adjustments. Because of the increasing interest in APMs in maternity care [12,13], it is important to understand which key design elements, and specifically which risk mitigation strategies, of APMs are used in current initiatives. As far as we are aware, such an overview of APMs in maternity care, as well as an overview of the available evidence on their effects on health and spending, is currently lacking. Therefore, this study aims to answer the following two research questions:

1. What are the key design elements of APMs currently implemented in maternity care?
2. What evidence is available with regard to the empirical effects of these alternative payment models on the maternal and neonatal health outcomes and perinatal spending?

## Methods

### Conceptual framework

Based on previous research [4,8,14,15], we developed a framework in which the key design elements of APMs are stated, to gain insight into their level of financial accountability for payers and providers and the level of integration of providers over domains of care. We defined APMs as initiatives that include changing the financing of care delivery that aimed to improve maternal and/or infant health outcomes and reduce utilization and/or health spending. Our definitions of the types of APMs are shown in ***Table 1***. Frakt and Mayes (2012) showed that the level of financial risk varies with the payment model. For example, under FFS, payers bear more risk than providers and under global payment providers bear more risk than payers [4]. Increased provider risk creates an incentive to collaborate with providers in other domains (as far as the included health services stretch) in order to reallocate and coordinate care efficiently. Yet, the level of financial risk a provider is bearing can be mitigated by ‘risk mitigation strategies’. Such risk mitigation strategies can lower (perceived) risks for providers in order to facilitate APM adoption or prevent providers from bearing too much risk (i.e. insurers’ risk). We distinguish between three types of risk mitigation strategies. First, strategies that are targeted at lowering provider risks via (sub)populations in the model. Examples are the exclusion of high-risk pregnancies and risk adjusted tariffs. Second, risk mitigation strategies that are targeted at the included services in the APM, such as a stop-loss provision (i.e. a threshold that caps the maximum amount to which the provider is at risk [16]) at the individual level. This is for example the exclusion of care delivered at a Neonatal Intensive Care Unit (NICU). Third, we distinguish between strategies that are aimed at reducing the total of risks that stem from the population and services, as for example a stop-loss provision at an aggregate level [17] or a risk corridor (with respect to losses) [18].

**Table 1:** APM types and definitions.


APM TYPE	DEFINITION

Pay-for-performance	In pay-for-performance, a bonus/malus is paid for attaining certain quality thresholds on top of the base FFS payment. Under FFS, providers are paid a fee for each service delivered [4]. The additional payments can be employed for improving coordination, care efficiency, quality of care or accessibility of care [19].

Shared savings	In a shared savings model, individual providers are each paid on a FFS basis which is combined with a reconciliation between the target episode price and the actual average episode price after a period of time across all the episodes attributed to a provider. Based on a specific formula, which is either negotiated or established by the payer, the accountable provider can share in gains and/or losses with the payer. Shared savings models that only share in gains are called *one-sided*. In *two-sided* models also incurred losses are shared.

Bundled payments	Bundled payments are defined for a specific set of activities tied to an episode of care, such as maternity care, that includes more than one provider or organization. The entity receiving the bundled payment earns a higher margin if a patient has utilized less care, but also bears the financial risk of complications. In our definition, the main difference with shared savings is that savings or losses are not shared with the payer. There are two types of bundled payments, retrospective and prospective. In *retrospective* bundled payments, there is a virtual budget negotiated upfront, providers are paid by FFS and retrospectively, the target price is reconciled [20]. In a *prospective* bundled payment model a prospectively defined prize is paid as one payment to the accountable entity that in turn pays the individual providers [14].

Global payments	In global payments the entire population and the entire continuum of care is included. The accountable provider is paid a fixed fee per head of the population.


APM: Alternative Payment Model; FFS: fee-for-service.

### Search strategy and information sources

To identify as many initiatives as possible that implemented APMs for maternity care, we reviewed the international literature. The process was guided by the Preferred Reporting Items for Systematic Reviews and Meta-Analysis (PRISMA) to ensure validity and reliability [21]. In collaboration with a librarian, we developed a search strategy for PubMed, Embase and Scopus databases for articles published from January 2007 through October 2020. Search key words included ‘payment’, ‘funding’, ‘alternative payment’, ‘value based payment’, ‘maternity ward’, ‘obstetric’, etc. The full search strategy is displayed in Appendix S1.

### Eligibility criteria

English or Dutch language articles were found eligible if they described and/or empirically evaluated, APMs in maternity care in high-income countries. Additional relevant articles and grey literature (e.g. government reports, white papers) were identified through reference tracking and recommendations from experts. Articles were excluded if they were commentary articles.

### Study selection

First, duplicates were removed. Based on title and abstract, the remaining articles were screened for eligibility by three researchers (EdV, ZS and MdBK) independently. Differences were discussed and, if there remained any doubt, the full-texts were retrieved to reach consensus on whether or not to include the article.

### Data extraction and synthesis

From the full-text articles, the following information was extracted: first author, year of publication, country, publication type, name of initiative and key design elements of the implemented APMs:

- Type of APM
- Care providers that participated in the model
- Accountable entity
- Care activities that are covered by the model
- Link of the model with quality of care.

From studies that empirically evaluated APMs, we additionally extracted information on the research method, data collection period and results of the payment model on health outcomes and healthcare spending. We assessed the quality of the evidence using the Effective Public Health Practice Project Quality Assessment Tool for Quantitative Studies [22]. This tool provides an overall methodological rating of the article: strong, moderate or weak based on assessment of six components (selection bias, study design, confounders, blinding, data collection methods and withdrawals and dropouts). The quality appraisal was performed by EdV and MdBK, independently. Discrepancies were resolved by discussion until consensus was reached. The evidence of the studies was not pooled. If blanks or uncertainty remained, the authors of the articles were requested to provide additional information through e-mail. EdV subtracted the data. JS and MdBK checked this randomly.

## Results

### Study selection and characteristics

**Figure 1:**
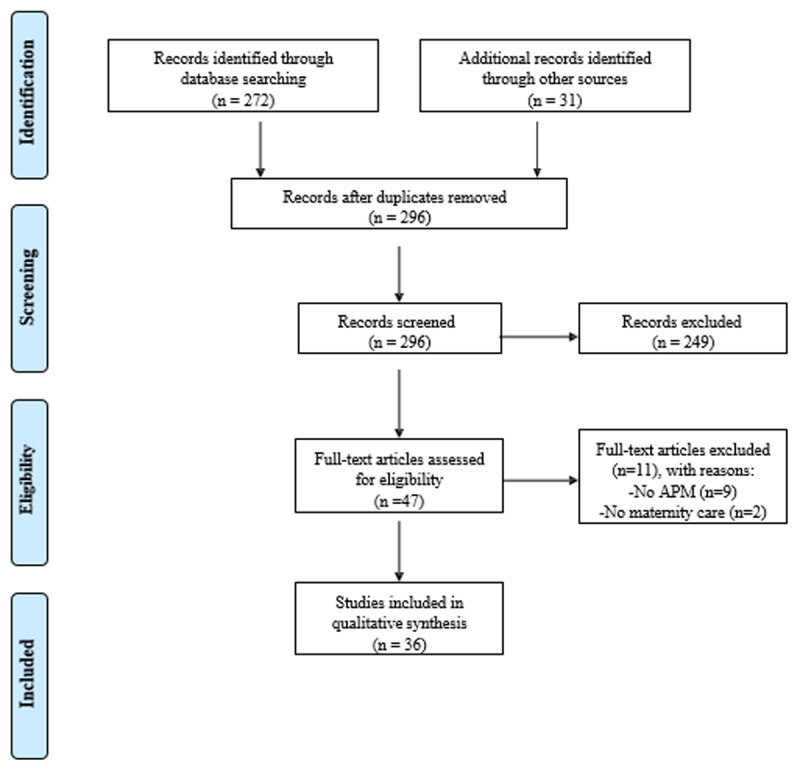
Study selection flow diagram according to Preferred Reporting Items for Systematic Review and Meta-Analysis (PRISMA).

***Figure 1*** shows the study selection flow diagram. We identified 272 articles through a search in the peer-reviewed international literature. Reference tracking yielded an additional 31 documents of which also non-scientific articles, white papers, government documents and blogs. After removal of duplicates and non-eligible articles, our final sample consisted of six articles from peer-reviewed journals and 30 government documents, white articles or other documents. Four peer-reviewed articles performed an empirical evaluation of the APM on health outcomes and health spending. The documents and document types included in this review are fully listed in Appendix S3.

### Key design elements of APMs in maternity care

#### General characteristics

In the 36 articles, we identified 17 initiatives that had implemented an APM (***Table 2***). Most of them are from the United States (n = 13) and further initiatives are found in the United Kingdom (n = 2), New Zealand (n = 1) and the Netherlands (n = 1). The earliest APMs were implemented in 2007 (GHS (16) and LMC (17)) and the most recent in 2017 (Dutch BP (13)). Most initiatives are established on a permanent basis (n = 11); five others were pilots, and for one the status is unknown.

**Table 2:** Key design elements of implemented alternative payment models in maternity care (more detailed information in Appendix S2).


NO	INITIATIVE (ABBREVIATION)	GENERAL CHARACTERISTICS			TYPE OF APM	INCLUDED POPULATION	INCLUDED CARE SERVICES	RISK MITIGATION STRATEGIES			REFERENCES
	
		COUNTRY	YEAR OF IMPLEMENTATION	STATUS				POPULATION	SERVICES	TOTAL EXPENDITURES	

1	Commissioning for Quality and Innovation, Payment Framework (CQUIN)	UK	2007	Permanent implementation	Pay for performance	Pregnancies ending in elective or emergency caesarean section	All perinatal hospital care	-	-	-	[23]

**2**	Texas Medicaid Program (Texas)	US (Texas, and later on also Georgia, Michigan, New Mexico, New York and South Carolina)	2011	Developed into initiative no. 3	Pay for performance	Penalty for neonatal delivery before 37 weeks gestation that was not medically necessary	n.a.	-	-	-	[24,25]

**3**	Horizon Blue Cross Blue Shield of New Jersey, Pregnancy Episodes of care program (Horizon)	US (New Jersey)	2013	Permanent implementation	Shared savings (one-sided).	Low-risk pregnancies (2013-present) and high-risk pregnancies (2015-present)	All prenatal outpatient care, all delivery-related care	Exclusion of pregnancies with comorbidities such as HIV and cancer.	Exclusion of neonatal care.	-	[25]

**4**	Baby+ Company	US (North Carolina, Tennessee, Colorado, Arkansas)	2013	Permanent implementation	Shared savings (one-sided).	Low-risk pregnancies	Care for mother and newborn	Exclusions of high-risk pregnancies	Exclusion of lab testing and ultrasounds	-	[17]

**5**	TennCare	US (Tennessee)	2013	Permanent implementation	Shared savings (two-sided)	Low- to medium-risk pregnancies with live births	Care for mother and newborn.	Exclusion based on clinical and cost-based criteria.	Exclusion of preconception care and neonatal care.	Total averages for benchmark for shared savings/losses are risk-adjusted	[26,27,28,29,30,31]

**6**	Arkansas Health Care Payment Improvement Initiative (Arkansas)	US (Arkansas)	2013	Permanent implementation	Shared savings (two-sided).	Low- to medium-risk pregnancies with live births	Care for mother and newborn	Exclusion based on clinical and cost-based criteria.	Exclusion of neonatal and preconception care.	Total averages for benchmark for shared savings/losses are risk-adjusted.	[17,27,32,33,34,35,36]

**7**	Ohio Episode-Based Payment Model (Ohio)	US (Ohio)	2015	Permanent implementation	Shared savings (two-sided).	Low- to medium-risk pregnancies with live births	Care for mother only	Exclusion based on clinical and cost-based criteria.	Exclusion of prenatal medications, neonatal care and preconception care.	Risk adjustment for the calculation of the shared savings /losses.	[17,27,30,37]

**8**	Community Health Choice, Bundled Payment Pilot (CHC)	US (Texas)	2015	Pilot	Shared savings (two-sided)	All pregnancies	Care for mother and newborn	Individual stop loss provision.	Exclusion of level 4 neonatal intensive care.	Risk adjustment	[17,38,39]

**9**	New York State’s Medicaid Maternity Care Value Based Payment Arrangement (New York)	US (New York state)	2016	Pilot	Shared savings (two-sided)	All pregnancies	Care for mother and newborn	Exclusion of mothers aged <12 or > 64 at the time of the delivery, maternal death, stillborn and multiple live births, HIV/aids or intellectually or developmentally disabled.	-	Risk adjustment	[40,41,42]

**10**	Pacific Business Group on Health, Blended Case Rate (Pacific)	US (Southern California)	2014	Pilot	Bundled payment (only delivery phase)	All hospital deliveries	All care activities during labor and delivery for both vaginal and caesarean section births.	Exclusion of pregnancies that left against medical advice, transferred during labor, various comorbidities (e.g. HIV/aids, cancer, also gestational age <37 weeks, multi gestation 3+). No prospective risk adjustment.	-	-	[17,33,43,44,45]

**11**	Minnesota Blended Payment (Minnesota BP)	US (Minnesota)	2009	Permanent implementation	Bundled payment (only delivery phase)	Uncomplicated births	Professional services and facility fees for vaginal or caesarean delivery and prenatal and postnatal care	Exclusion of complicated vaginal deliveries	-	-	[46,47]

**12**	Minnesota Birth Centers, BirthBundle (BirthBundle)	US (Minnesota)	2015	Pilot stopped	Bundled payment (retrospective).	All pregnancies	Care for mother and newborn	-	-	-	[17,48]

**13**	Bundled Payment for maternity care (The Dutch BP)	The Netherlands	2017	Pilot	Bundled payment (prospective)	All pregnancies	Bundle defined in four phases (prenatal, natal, postnatal and postnatal home assistance), covering all necessary care - according to the national care standardization guidelines	Risk stratification for bundled tariff.	-	Depending on the contract, risk corridors on where new negotiations are started - are negotiated.	[49,50,51,52]

**14**	Maternity Pathway Bundled Payment (Maternity Pathway BP)	England	2013	Permanent implementation	Bundled payment (prospective)	All pregnancies	All maternal and neonatal care in the prenatal, perinatal and postpartum phase	Risk stratification for bundle tariff.	Exclusion of health problems in neonates	-	[53]

**15**	Providence Health and Services, Pregnancy Care Package (Providence)	US (Oregon)	2013	Permanent implementation	Bundled payment (prospective)	Low-risk pregnancies	Care for mother and newborn	-	-	-	[17]

**16**	Geisinger Health System, Perinatal ProvenCare Initiative (GHS)	US (Pennsylvania)	2007	Permanent implementation	Bundled payment (prospective)	Low-risk pregnancies	Care for mother only	Exclusion of late referrals and high risk pregnancies	Exclusion of neonatal care	-	[17,33,54]

**17**	Lead Maternity Care Model (LMC)	New Zealand	2007	Permanent implementation	Bundled payment (prospective)	All pregnancies	Care for mother only	-.	Exclusions of neonatal care and the consultation of obstetricians.	-	[55,56,57]


#### Type of APM

***Table 2*** also shows the type of APM of the 17 initiatives. The APMs are classified into three categories: pay-for-performance (n = 2), shared savings models (n = 7) and bundled payment models (n = 8). For a detailed overview including all key design elements (type of APM, care providers that participated in the model, accountable entity, care activities that are covered by the model and link of the model with quality of care), see Appendix S2.

Pay-for-performance is applied in two initiatives (1,2). In the CQUIN initiative in England (1), hospitals are paid bonuses if they satisfy specified scores on a set of quality indicators pertaining to elective and emergency Caesarean sections. This pay-for-performance system is superimposed onto the existing FFS model, hence not replacing the existing payment structure. In the Texas Medicaid Program (2) there is a penalty for hospitals that performed neonatal deliveries before 37 weeks gestation that are not medically necessary; these billing codes are ineligible for reimbursement.

Shared savings models are implemented in seven initiatives (Horizon (3), Baby+Company (4), TennCare (5), Arkansas (6), Ohio (7), CHC (8) and New York (9)). Those are contracts in which any achieved savings are shared between providers and payers. Such savings are calculated by comparing the health care spending for the risk adjusted population included in the payment model either with the spending for a predefined control group (i.e. concurrent accountable providers) (TennCare (5), Arkansas (6), Ohio (7)) or with the spending for the intervention population in years preceding the implementation of the APM (historical benchmark) (Horizon (3), CHC (8)). If savings are achieved for the intervention population in comparison with the control group or the historical benchmark, those savings are partially distributed to the providers, resulting in for example 50% for the providers and 50% for the payers (TennCare (5)).

The shared savings contracts vary in the degree to which health care providers bear financial risks in the event of spending overruns. Two initiatives, Horizon (3) and Baby+Company (4), operated a one-sided shared savings model, in which providers bear no downside risks if budgets are exceeded but share in any savings achieved. Five initiatives, TennCare (5), Arkansas (6), Ohio (7), CHC (8) and New York (9), agreed in their contracts that the providers must reconcile any spending overruns (two-sided models). In the more recently launched initiatives CHC (8) and New York (9), the shared savings contracts employed one-sided models in the first year but were converted to two-sided contracts in the second year, thus gradually shifting more financial risks towards the providers. Four of the shared savings models (Horizon (3), TennCare (5), Arkansas (6) and Ohio (7)) appoint the provider (group) that delivers the baby as the accountable entity. In the shared savings models, the distribution of savings is contingent on achieved improvements in quality. In the New York scheme (9), provider penalties for exceeding budgeted spending are reduced or eliminated for those scoring high on the quality indicators.

Eight initiatives (10, 11, 12, 13, 14, 15, 16, 17) implemented variants of bundled payment models that vary highly in terms of shifting accountability. Two initiatives (Pacific (10) and Minnesota BP (11)) only cover care in the delivery phase for which the hospital is accountable. A fixed fee is negotiated for deliveries, irrespective of whether vaginal or Caesarean. In the Minnesota BP (11) program, complicated vaginal deliveries are excluded. Although the payment model is limited to the hospital (i.e. one provider only), this model contains a financial incentive to perform fewer Caesarean sections. Therefore, one may conclude that the financial risk in this model is partially shifted from the payer to the care provider(s). That is why we decided to classify this model as an APM and included it in our overview.

One initiative (BirthBundle (12)) implemented a retrospective model. The integrated fee that is charged for a maternity care episode is in fact a ‘virtual’ fee, which is reconciled at the end of the episode by totaling the FFS for all the services delivered. If the spending turned out lower or higher than the virtual fee, the difference is transferred to the accountable entity, which is the birth center (BirthBundle (12)).

Five initiatives (The Dutch BP (13), Maternity Pathway BP (14), Providence (15), GHS (16) and LMC (17)) implemented prospective bundled payment models. All the services specified in the entire maternity care program (or split up into three or four phases (prepartum, delivery and postpartum) (The Dutch BP (13), Maternity Pathway BP (14), LMC (17)) are contracted, delivered and claimed as a single product by the accountable nurse or midwife or obstetrician. These prospective bundled payment models replaced the existing FFS models. No reconciliations are performed as in the retrospective models.

#### Included population, included care services and risk mitigation strategies

The pay-for-performance models (n = 2) employ no risk mitigation strategies.

In the one-sided shared savings models (n = 2) risk mitigation strategies are targeted at the population and the services included in the model. Baby+Company (4) only includes low-risk pregnancies and the Horizon program (3) includes both low- and high-risk pregnancies, but excludes several comorbidities in pregnancy such as HIV and cancer and neonatal care to set the benchmark. Baby+Company (4) excludes lab testing and ultrasounds. Both one-sided shared savings models include care for the mother and the newborn. Risk mitigation strategies at the level of total expenditures were not found for these shared savings models.

The two-sided shared savings models (n = 5) do employ risk mitigation strategies at the level of total expenditures. The benchmarks for savings and losses are risk adjusted in all initiatives. At the population and the services level, TennCare (5), Arkansas (6), Ohio (7) and CHC (8) include a stop-loss provision for individual cases that exceed the risk-adjusted mean by more than three times the standard deviation. They include only low-risk pregnancies and exclude pregnancies ending in stillbirth. In addition, women with several comorbidities are excluded from the model, as is preconception and neonatal care. New York (9) includes all pregnancies and care for the newborn, but excludes mothers aged under 12 or above 64, maternal death, stillborn and multiple live births. Also mothers with HIV/aids and mothers who are intellectually or developmentally disabled are excluded in the New York (9) model.

For the bundled payment models focusing only on the delivery phase (n = 2), only risk mitigation strategies on the population level were found. Pacific (10) excludes various comorbidities and cases that left against medical advice or were transferred during labor. Minnesota BP (11) excludes complicated vaginal deliveries.

For the retrospective bundled payment model (n = 1), BirthBundle (12), we did not find any risk mitigation strategies.

The prospective bundled payment models (n = 5) apply different forms of risk mitigation strategies. The Dutch BP (13), Maternity Pathway BP (14) and LMC (17) include low- and high-risk pregnancies. Providence (15) and GHS (16) only include low-risk pregnancies. The Maternity Pathway BP (14) and the Providence (15) initiative include care for the newborn as well as the mother, although health problems of newborns are excluded in the Maternity Pathway BP (14). GHS (16) and LHC (17) only include care services for the mother and not for the newborn. GHS (16) excludes late referrals or members not enrolled at least 12 continuous weeks of the prenatal phase. The Dutch BP (13) and Maternity Pathway BP (14) apply risk based tariffs. In the Netherlands (13), at the aggregated level, in most regions, risk corridors are applied. Other risk mitigation strategies were not found. As a quality assurance measure with respect to the care delivered, payers generally required providers to file yearly reports on quality indicators (accountability data).

**Figure 2:**
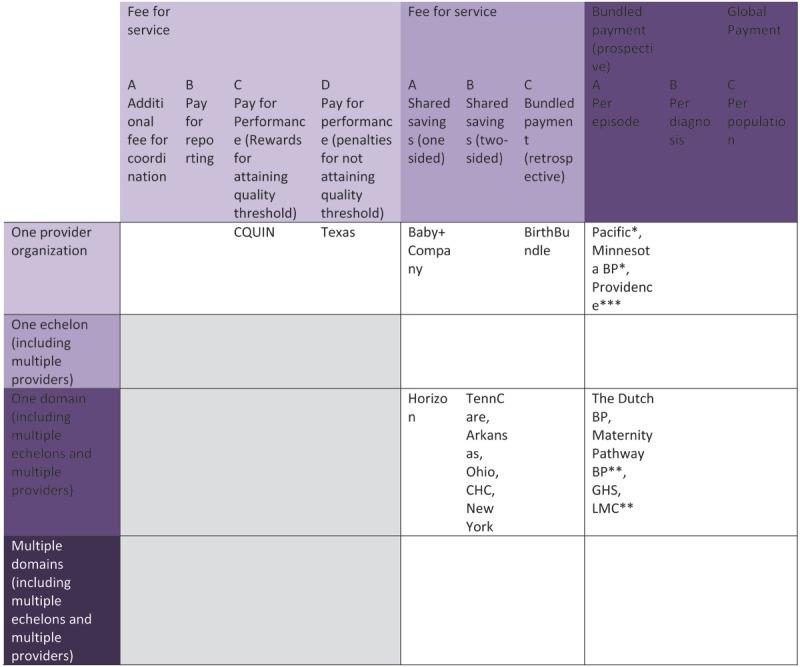
Level of integration and financial accountability of the alternative payment models in maternity care. *episode is limited to the delivery phase in the hospital only, **episode is divided into three or four phases (prenatal, delivery, postnatal, maternity community care), ***integrated birth center; CQUIN: Commissioning for Quality and Innovation Payment Framework; Texas: Texas Medicaid Program; BirthBundle: Minnesota Birth Centers BirthBundle; Pacific: Pacific Business Group on Health Blended Case Rate; Minnesota BP: Minnesota Blended Payment; Providence: Providence Health and Services Pregnancy Care Package; Horizon: Horizon Blue Cross Blue Shield of New Jersey, Pregnancy Episodes of care Program; Arkansas: Arkansas Health Care Payment Improvement Initiative; Ohio: Ohio Episode-Based Payment Model; CHC: Community Health Choice, Bundled Payment Pilot; New York: New York States’ Medicaid Maternity Care Value Based Payment Arrangement; The Dutch BP: The Dutch Bundled Payments for Maternity Care; Maternity Pathway BP: Maternity Pathway Bundled Payment; GHS: Geisinger Health System Perinatal ProvenCare Initiative; LMC: Lead Maternity Carer.

***Figure 2*** provides a schematic overview of the various payment models, showing the level of integration and the level of financial accountability. APMs that employ the highest level of integration and highest level of financial accountability are the Maternity Pathway Bundled Payment, GHS and LMC.

### Effects of the APMs on maternal and neonatal health outcomes and health spending

***Table 3*** shows results of the available evaluations from four of the 17 initiatives that implemented an APM in maternity care (Texas (2), Arkansas (6), Minnesota BP (11) and GHS (16)). In two studies, a beneficial effect of the APM (Texas (2) and GHS (16)) on the health outcomes was observed [24,54]. The other two studies that evaluated the effects of the APMs [32,46] did not show improvement on health outcomes for Arkansas (6) and Minnesota BP (11). Two studies [32,46] gauged the effects of the scheme (Arkansas (6) and Minnesota BP (11)) on health care expenditures, reporting positive effects.

**Table 3:** Effects of the alternative payment models in maternity care on health outcomes and health spending.


INITIATIVE (ABBREVIATION)	TYPE OF APM	STUDY DESIGN	DATA COLLECTION PERIOD	RESULTS – HEALTH OUTCOMES	RESULTS – SPENDING	QUALITY APPRAISAL OF THE STUDY	REFERENCES

Geisinger Health System, Perinatal ProvenCare Initiative (GHS)	Bundled payment (prospective)	Observational study design. Pre-intervention period (n = 101); post-intervention period (n = 1,010)	Pre-intervention population: January 2008 – October 2008, Post-intervention population: April 2009 – June 2010.	Improvement on nearly all 103 indicators: e.g. 25% reduction in neonatal intensive care admissions and screening and prevention activities for smoking increased from 45% to 88%.	-	Weak	[54]

Arkansas Health Care Payment Improvement Initiative (Arkansas)	Shared savings (two-sided)	Difference-in-difference design. Pre-intervention: n = 2,454 (intervention); n = 20,824 (controls). Post-intervention: n = 1,737 (intervention); n = 15,291 (controls)	Pre-intervention: 2010 to 2012, Post-intervention: 2013 - 2014	-	Perinatal spending decreased by 3.8% overall. The decrease was driven by the prices paid for inpatient facility care.	Moderate	[32]

Minnesota Blended Payment (Minnesota BP)	Bundled payment (only delivery phase)	Interrupted time series design. Experiment group n = 25,080; Control group n = 646,097	2006–2012	There were no significant effects on maternal morbidity.	Spending dropped by $425.80. It continued to decrease in by $95.04 per quarter.	Moderate	[46]

Texas Medicaid Program (Texas)	Pay-for-performance	Difference-in-difference design. Experiment group n = 438,429. Control group 1 n = 895,543; control group 2 n = 1,691,896; control group 3 n = 573,382	2009–2013	Gains of five days in gestational age and six ounces in birthweight.	-	Moderate	[24]


Three out of four studies reporting evaluations were assigned moderate rating for the quality of the evidence. No studies evaluating APM in maternity care received a strong rating. Appendix S4 summarizes the details on quality of the evidence assessments that we conducted.

## Discussion

In order to enhance APM adoption more insight in the key design elements, including risk mitigation strategies, of APMs is essential. Risk mitigation strategies can be helpful to increase APM uptake and protect providers from bearing too much (perceived) financial risk. In addition, insights in the empirical evidence of APMs on maternal and infant health outcomes and spending are needed. We identified 17 initiatives implementing APMs in maternity care: pay-for-performance models (n = 2), shared savings models (n = 7) and bundled payment models (n = 8). APMs that shifted more financial accountability towards providers, like bundled payments for example, seemed to include more strategies that mitigated those risks. Risk mitigation strategies included population and care exclusions, stop loss provisions and risk adjustment. Preliminary evaluations of APMs (n = 4) showed either positive effects or no effect on health outcomes and spending. Although these first studies examining the effects of APMs on health outcomes and health spending in maternity care seem tentatively positive, extensive conclusions on the effects of the APMs cannot be drawn.

These either positive or no observed effects in the preliminary evaluations of APMs are remarkable, as in most models, a substantial part of the intended exposure (i.e. bearing financial risks) has been mitigated by the applied risk mitigation strategies. Without these risk mitigation strategies, the incentive would have been greater, which may have led to greater observed effects. This notion could encourage providers to adopt APMs, as the lack of observed negative effects seems to make it a rather safe bet. In general, risk mitigation strategies seem to play an important role in increasing the uptake of APMs by limiting the (perceived) risks for providers. These strategies should however be applied in moderation or for a limited period of time only, otherwise the (theoretical) incentives of the APM are subverted by eliminating the risks that are needed to create these incentives. Such a stepwise increase of financial risks borne by the providers is already applied in more recently launched initiatives (such as CHC (8) and New York (9)).

To deal with the current demand for payment reform [12], and in the absence of conclusive evidence on the effects of APMs, we identified two issues that should be addressed in order to design an APM that fits the health care setting at hand and works towards the desired goals.

First, a detailed understanding of the specific elements of the APMs is required with providers and payers that work to implement APMs. In this review, we found that currently there is a multiplicity of complex terminology and ambiguous definitions that confuses the understanding of APMs. For example, we found that descriptions of APMs often used the terms ‘shared savings’ and ‘bundled payments’ rather interchangeably. That was notably the case in initiatives that employed a two-sided shared savings model or a retrospective bundled payment model. Presumably, the conflation of the two notions arises from the conceptual similarities between the two payment models. However, the proportion of risk borne by the care providers working under the model is 100% in bundled payment contracts, and it is smaller (for instance a 50–50% split or a 30–70% split) in shared savings contracts. A distinction like this for example was not clearly made in several of the descriptions, and as such confusing the two terms. A clear definition of terminology will contribute to a better understanding of the key design elements of the APMs.

Second, key design elements of the APM including risk mitigation strategies may be best designed from the providers’ perspective. For example, feasibility considerations may play an important role in designing the APM. We found, for example, that in some initiatives the APM had been superimposed onto the existing FFS model for the single reason that retrospective reconciliation is easier to administer within the current FFS environment (also see [58]). This aligns with the theoretical notion that the ‘best’ APM shifts only that part of the accountability towards the provider that actually can be influenced by the provider. Careful deliberation that is based on the level of accountability the provider is willing and able to bear, should lead to which type of APM and which types of risk mitigation strategies, are the most optimal to apply in order to deliver optimal care for the best attainable health for mothers and children. Consequently, there should be a shift from current insurer-tailored contracts towards provider-tailored contracts to facilitate the adoption of APMs.

Limitations of this study include that we might have missed relevant initiatives implementing APMs in maternity care by, for example, the use of different terminology. Nevertheless, as the aim was to provide an exhaustive list of these initiatives, we deliberately chose to use other sources than peer-reviewed journals, such as government documents and white papers. Therefore, we are confident that we captured the majority of the initiatives implementing APMs in maternity care in high-income countries. Another limitation is that we did not include grey literature in the search for the evaluations on the effects of APMs in maternity care. Although we may have missed relevant insights in the effects of APMs on health outcomes and spending, we are convinced that we were able to assess the quality of the included studies by using the Effective Public Health Practice Project Quality Assessment Tool for Quantitative Studies (on peer-reviewed evaluation studies only).

### Conclusions and implications

We identified maternity care APMs in the United States, New Zealand, United Kingdom and the Netherlands. All such APMs intended to improve health outcomes and reduce the spending level of maternity care by shifting financial accountability from payers to providers. At the same time, APMs that shifted more financial accountability towards providers seemed to include more strategies that mitigated those risks. Although first evaluations of APMs in maternity care seem tentatively positive, due to a variety of model elements and health system characteristics they operate in, extensive or general conclusions could not be drawn. Further research, clearly defining the different key design elements and an in-depth understanding of these key design elements, as well as providing insights in the effects of APMs under the influence of unique characteristics of health systems, is required to understand future evidence and shape payment reform that aligns with its goals.

## Additional File

The additional file for this article can be found as follows:

10.5334/ijic.5535.s1 Appendix S1. Search strategy.

10.5334/ijic.5535.s2 Appendix S2. Detailed characteristics of the 17 initiatives employing APMs in maternity care.

10.5334/ijic.5535.s3 Appendix S3. Full-text document types.

10.5334/ijic.5535.s4 Appendix S4. Quality appraisal of the studies performing an effect evaluation of an APM in maternity care.
