# Supplementary material for: A Scoping Review of Alternative Payment Models in Maternity Care: Insights in Key Design Elements and Effects on Health and Spending
Source: Int J Integr Care. 2021 Apr 21;21(2):6. doi: 10.5334/ijic.5535 (PMC8086739; doi:10.5334/ijic.5535)
Supplement: Appendix S1. — Search strategy. [file ijic-21-2-5535-s1.pdf]

Appendix S1 – search strategy

Embase session results (comparable strategy voor Scopus and Pubmed)

105
#16
#15 AND [1-12-2016]/sd

125
#15
#14 AND [2016-2019]/py

797
#14
#13 NOT ('**asia**'/exp OR '**africa**'/exp OR '**south and central america**'/exp)

937
#13
#12 AND (**english**:la OR **dutch**:la)

982
#12
#10 NOT **#11**

5,901,894
#11
letter:it OR **note**:it OR **erratum**:it OR **news**:it OR '**conference abstract**':it OR '**conference paper**':it OR '**conference review**':it

1,326
#10
#3 AND (**#4** OR **#5** OR **#6** OR **#7** OR **#8** OR **#9**)

29,268
#9
'**new zealand**'/mj OR '**new zealand**':ti

64,271
#8
'**prospective payment**'/mj OR '**bundled payment**'/mj OR '**reimbursement**'/mj OR '**healthcare financing**'/mj OR '**financing, organized**'/mj OR '**financing, government**'/mj OR ('**integrated health care system**'/de AND **pay***:ti,ab)

156
#7
'**individual funding**':ti,ab OR '**personal funding**':ti,ab OR '**individual budget***':ti,ab OR '**personal budget***':ti,ab

427
#6
'value based reimbursement':ti,ab OR **'funding model'**:ti,ab OR **'integrated funding model*'**:ti,ab OR **'case rate payment*'**:ti,ab OR **'individualised funding'**:ti,ab OR **'individualized funding'**:ti,ab OR **'personalised funding'**:ti,ab OR **'personalized funding'**:ti,ab OR **'individualised budget*'**:ti,ab OR **'individualized budget*'**:ti,ab OR **'personalised budget*'**:ti,ab OR **'personalized budget*'**:ti,ab

673
#5
((**bundle*** NEAR/5 **pay***):ti) OR ((**episode*** NEAR/5 **pay***):ti,ab)

45,105
#4
((**payment*** NEAR/3 **model***):ti,ab) OR ((**alternative** NEAR/3 **payment***):ti,ab) OR **'funding source*'**:ti,ab OR **'funding strateg*'**:ti,ab OR **'funding system*'**:ti,ab OR **'service funding'**:ti,ab OR **'financing source*'**:ti,ab OR **'financing strateg*'**:ti,ab OR **'financing system'**:ti,ab OR ((**pay** NEAR/3 **expenses**):ti,ab) OR **'bear cost*'**:ti,ab OR **'payment system*'**:ti,ab OR **reimbursement**:ti,ab OR **'payment reform*'**:ti,ab OR **'payment policy change*'**:ti OR **payment***:ti

308,705
#3
#1 OR **#2**

303,021
#2
'maternity ward'/de OR **'obstetric delivery'**/de OR **'home delivery'**/de OR **'obstetrics'**/de OR **'obstetrical nursing'**/de OR **'nurse midwifery'**/de OR **'midwife'**/de OR **'newborn nursing'**/de OR **'neonatal nurse practitioner'**/de OR **'perinatal care'**/de OR **'perinatal nursing'**/de OR **'perinatal mortality'**/de OR **'perinatal outcome'**/de OR **'postnatal care'**/de OR **'newborn care'**/de OR **'newborn assessment'**/de OR **'newborn death'**/de OR **'newborn intensive care'**/de OR **'newborn intensive care nursing'**/de OR **'newborn intensive care unit'**/de OR **'newborn monitoring'**/de OR **'maternal care'**/de OR **'maternal health service'**/de OR **'prenatal care'**/de OR **'prenatal diagnosis'**/de OR **'prepregnancy care'**/de OR **'pregnancy outcome'**/de

25,175
#1
'birthing centers':ti OR **'birthing centres'**:ti OR **'obstetric care'**:ti OR **'obstetric services'**:ti OR **'obstetric health services'**:ti OR **midwifery**:ti OR **midwives**:ti OR **'birth attendants'**:ti OR **deliveries**:ti OR **'prenatal care'**:ti OR **'antenatal care'**:ti OR **'natal care'**:ti OR **'birth care'**:ti OR **'neonatal care'**:ti OR **'perinatal care'**:ti OR **'perinatal health'**:ti OR **'perinatal services'**:ti OR **'perinatal population*'**:ti OR **'perinatal outcome*'**:ti OR **'maternal care'**:ti OR **'maternal health services'**:ti OR **'maternal services'**:ti OR **'maternity care'**:ti OR **'pregnancy care'**:ti OR **'pregnancy services'**:ti OR **'pre-pregnancy care'**:ti OR **'prepregnancy care'**:ti OR **'pregnancy related services'**:ti OR **homebirth***:ti

Section/topic	#	Checklist item	Reported on page #
**TITLE**			
Title	1	Identify the report as a systematic review, meta-analysis, or both.	1
**ABSTRACT**			
Structured summary	2	Provide a structured summary including, as applicable: background; objectives; data sources; study eligibility criteria, participants, and interventions; study appraisal and synthesis methods; results; limitations; conclusions and implications of key findings; systematic review registration number.	3
**INTRODUCTION**			
Rationale	3	Describe the rationale for the review in the context of what is already known.	5/6
Objectives	4	Provide an explicit statement of questions being addressed with reference to participants, interventions, comparisons, outcomes, and study design (PICOS).	6 (n/a)
**METHODS**			
Protocol and registration	5	Indicate if a review protocol exists, if and where it can be accessed (e.g., Web address), and, if available, provide registration information including registration number.	6
Eligibility criteria	6	Specify study characteristics (e.g., PICOS, length of follow-up) and report characteristics (e.g., years considered, language, publication status) used as criteria for eligibility, giving rationale.	6
Information sources	7	Describe all information sources (e.g., databases with dates of coverage, contact with study authors to identify additional studies) in the search and date last searched.	6
Search	8	Present full electronic search strategy for at least one database, including any limits used, such that it could be repeated.	6
Study selection	9	State the process for selecting studies (i.e., screening, eligibility, included in systematic review, and, if applicable, included in the meta-analysis).	7
Data collection process	10	Describe method of data extraction from reports (e.g., piloted forms, independently, in duplicate) and any processes for obtaining and confirming data from investigators.	7
Data items	11	List and define all variables for which data were sought (e.g., PICOS, funding sources) and any assumptions and simplifications made.	7/8

Risk of bias in individual studies	12	Describe methods used for assessing risk of bias of individual studies (including specification of whether this was done at the study or outcome level), and how this information is to be used in any data synthesis.	8
Summary measures	13	State the principal summary measures (e.g., risk ratio, difference in means).	n/a
Synthesis of results	14	Describe the methods of handling data and combining results of studies, if done, including measures of consistency (e.g., I^2) for each meta-analysis.	n/a

Section/topic	#	Checklist item	Reported on page #
Risk of bias across studies	15	Specify any assessment of risk of bias that may affect the cumulative evidence (e.g., publication bias, selective reporting within studies).	9, 14/15
Additional analyses	16	Describe methods of additional analyses (e.g., sensitivity or subgroup analyses, meta-regression), if done, indicating which were pre-specified.	n/a
**RESULTS**			
Study selection	17	Give numbers of studies screened, assessed for eligibility, and included in the review, with reasons for exclusions at each stage, ideally with a flow diagram.	8/9
Study characteristics	18	For each study, present characteristics for which data were extracted (e.g., study size, PICOS, follow-up period) and provide the citations.	9 and table 2
Risk of bias within studies	19	Present data on risk of bias of each study and, if available, any outcome level assessment (see item 12).	12/13
Results of individual studies	20	For all outcomes considered (benefits or harms), present, for each study: (a) simple summary data for each intervention group (b) effect estimates and confidence intervals, ideally with a forest plot.	n/a
Synthesis of results	21	Present results of each meta-analysis done, including confidence intervals and measures of consistency.	n/a
Risk of bias across studies	22	Present results of any assessment of risk of bias across studies (see Item 15).	14/15
Additional analysis	23	Give results of additional analyses, if done (e.g., sensitivity or subgroup analyses, meta-regression [see Item 16]).	n/a
**DISCUSSION**			
Summary of evidence	24	Summarize the main findings including the strength of evidence for each main outcome; consider their relevance to key groups (e.g., healthcare providers, users, and policy makers).	13
Limitations	25	Discuss limitations at study and outcome level (e.g., risk of bias), and at review-level (e.g., incomplete retrieval of identified research, reporting bias).	14/15

Conclusions	26	Provide a general interpretation of the results in the context of other evidence, and implications for future research.	15
**FUNDING**			
Funding	27	Describe sources of funding for the systematic review and other support (e.g., supply of data); role of funders for the systematic review.	1

From: Moher D, Liberati A, Tetzlaff J, Altman DG, The PRISMA Group (2009). Preferred Reporting Items for Systematic Reviews and Meta-Analyses: The PRISMA Statement. PLoS Med 6(7): e1000097. doi:10.1371/journal.pmed1000097

For more information, visit: **www.prisma-statement.org**.
