# Supplementary material for: A Scoping Review of Alternative Payment Models in Maternity Care: Insights in Key Design Elements and Effects on Health and Spending
Source: Int J Integr Care. 2021 Apr 21;21(2):6. doi: 10.5334/ijic.5535 (PMC8086739; doi:10.5334/ijic.5535)
Supplement: Appendix S2. — Detailed characteristics of the 17 initiatives employing APMs in maternity care. [file ijic-21-2-5535-s2.pdf]

Appendix S2 – Detailed characteristics of the 17 initiatives employing APMs in maternity care

No.	Initiative (abbreviation)	General characteristics			Type of APM							Risk mitigation strategy	Ref
		Country	Year of implementation	Status	Type of APM	Care providers participating in the payment model	Accountable entity	Eligible population	Episode time span	Care activities covered by payment model	Linkage to quality		
1	Commissioning for Quality and Innovation, Payment Framework (CQUIN)	United Kingdom	2007	Permanent implementation	Pay for performance	Single organisation	Hospital	Pregnancies ending in elective or emergency Caesarean section.	Delivery	All perinatal hospital care.	Yes: proportion of NHS providers income linked to achievement of specified thresholds on specified self-defined quality indicators	Unclear	[28]
2	Texas Medicaid Program (Texas)	US (Texas, and later also Georgia, Michigan, New Mexico, New York and South Carolina)	2011	Developed into initiative no. 3	Pay for performance	Single organisation	Hospital	Penalty for neonatal delivery before 37 gestation that was not medically necessary	Delivery	n.a.	n.a.	Unclear	[22, 29]
3	Horizon Blue Cross Blue Shield of New Jersey, Pregnancy Episodes of care program (Horizon)	US (New Jersey)	2013	Permanent implementation	Shared savings, upside only. Benchmark based on historical data.	Multiple organisations representing different levels of care	Provider	Low-risk pregnancies and high-risk pregnancies from 2015.	Till 30 days after birth	All prenatal outpatient care, all delivery-related care (vaginal/cs), postpartum care not known.	n.a.	Exclusion of pregnancies with comorbidities such as HIV and cancer. Exclusion of neonatal care.	[29]
4	Baby+ Company	US (North Carolina, Tennessee, Colorado,	2013	Permanent implementation	Shared savings (one-sided). Separate rates if transferred	Single health care organisation	Free standing birth center.	Low-risk pregnancies.	From initial obstetrics consultation at birth	Mother and newborn. Prenatal care including birthing plan, classes,	Yes, but method unclear	Exclusion of members not enrolled during entire	[19]

No.	Initiative (abbreviation)	General characteristics			Type of APM							Risk mitigation strategy	Ref
		Country	Year of implementation	Status	Type of APM	Care providers participating in the payment model	Accountable entity	Eligible population	Episode time span	Care activities covered by payment model	Linkage to quality		
		Arkansas)			before/during labor.				centre to 6 weeks postpartum	postpartum care, newborn exam, metabolic screen and medications. Includes facility and professional fees.		pregnancy, high-risk pregancies and complicated deliveries. Exclusions of lab testing and ultrasounds.	
**5**	TennCare	US (Tennessee)	2013	Permanent implementation	Shared savings (two-sided): spli 50/50	Multiple organisations representing different levels of care	Principle Accountable Provider is the healthcare provider who delivers the neonate	Low- to medium-risk pregnancies with live births	40 weeks before delivery to 60 days postpartum	All inpatient and outpatient medical services as well as ancillary services such as pharmacy and imaging services. All related prenatal care and medication or emergency department visits. All care during delivery. Postpartum care till 30 days: non-inpatient readmissions, non-admission ED care, other covered medication, practitioner and facility costs. Postpartum care till 60 days: all postpartum-related medical care and medications.	Yes: distribution of savings contingent on quality improvements achieved	Exclusion of episode based on clinical and cost-based exclusion criteria. Total averages for benchmark for shared savings/losses are risk-adjusted based on comorbidities, demographics and other indicators of patient variation. Exclusions, preconception and neonatal care.	[10, 30-34]
**6**	Arkansas Health Care Payment Improvement Initiative (Arkansas)	US (Arkansas)	2013	Permanent implementation	Shared savings (two-sided). PAPs may be rewarded, penalized, or remain financially neutral based on	Multiple organisations representing different levels of care	Principle Accountable Provider is the provider or provider	Low- to medium-risk pregnancies with live births	40 weeks before delivery to 60 days postpartum	All inpatient and outpatient medical services as well as ancillary services such as pharmacy and imaging services. All	Yes: distribution of savings contingent on quality improvemen	Exclusion of members not enrolled during the entire pregnancy	[10, 11, 19, 23, 35-]

No.	Initiative (abbreviation)	General characteristics			Type of APM							Risk mitigation strategy	Ref
		Country	Year of implementation	Status	Type of APM	Care providers participating in the payment model	Accountable entity	Eligible population	Episode time span	Care activities covered by payment model	Linkage to quality		
					how the average costs for their episodes compare with thresholds predetermined by payers.		group that performs the delivery.			prenatal care, care related to labor and delivery, postpartum maternal care.	ts achieved	period and of episodes based on clinical and cost-based exclusion criteria. Total averages for benchmark for shared savings/losses are risk-adjusted based on comorbidities, demographics and other indicators of patient variation. Cost saving payments are capped beyond a limit determined by each payer. Exclusions: costs not related to maternity care, neonatal care and preconception care.	[37]
**7**	Ohio Episode-Based Payment Model (Ohio)	US (Ohio)	2015	Permanent implementation	Shared savings (two-sided). Historically determined threshold.	Multiple organisations representing different levels of care	Principle Accountable Provider, which is the healthcare	Low- to medium-risk pregnancies with live births,	280 days before delivery to 60 days postpartu	Mother only. All inpatient and outpatient medical services as well as ancillary services such	Year 1: Yes: distribution of savings contingent on quality	Exclusion of members not enrolled for the entire pregnancy or	[10, 19, 33, 38]

No.	Initiative (abbreviation)	General characteristics			Type of APM							Risk mitigation strategy	Ref
		Country	Year of implementation	Status	Type of APM	Care providers participating in the payment model	Accountable entity	Eligible population	Episode time span	Care activities covered by payment model	Linkage to quality		
							provider who delivers the neonate		m	as pharmacy and imaging services, including office visits, supportive services such as psychosocial evaluation, counseling, eduction (e.g. breat feeding), vaginal delivery or caesarean sections, induction, epidural, additional screening, neonatal support in the delivery period, transportation, follow-up visits for the mother, readmission, postpartum depression, and genetic screening.	improvements achieved and the reporting of data on specified indicators, with threshold designed to make 75% of providers eligible for bonuses. From year 2: Yes: distribution of savings contingent on quality improvements achieved and the reporting of data on specified indicators, with threshold designed to make the highest quartile of providers eligible for bonuses	episodes based on clinical and cost-based exclusion criteria. Risk adjustment for the calculation of the shared savings /losses. Exclusions: prenatal medications, neonatal care and preconception care.	
**8**	Community Health Choice,	US (Texas)	2015	Pilot	Year 1: Shared savings (one-sided), split	Multiple organisations representing	Obstetricians from multispecial	Low-risk and high-risk pregnancies.	270 days before delivery to	Newborn. Three components: pregnancy, delivery	Year 1: Yes: Providers were to	Risk adjustment based on risk	[19, 39,]

No.	Initiative (abbreviation)	General characteristics			Type of APM							Risk mitigation strategy	Ref
		**Country**	**Year of implementation**	**Status**	**Type of APM**	**Care providers participating in the payment model**	**Accountable entity**	**Eligible population**	**Episode time span**	**Care activities covered by payment model**	**Linkage to quality**		
	Bundled Payment Pilot (CHC)				50/50. Year 2: shared savings (two-sided), split (greater for gains for providers, smaller for providers for losses). Benchmark based on historical average costs adjusted with yearly growth trends. In three components; pregnancy, delivery and newborn.	different levels of care	ty provider groups.		60 days postpartum	and newborn care. All prenatal care and services related to delivery (with blended rate for vaginal and Caesarean delivery). Postpartum: maternal and neonatal care (levels 1 to 3) till 30 days postpartum.	report accountability data, though outcomes had no financial consequences. Year 2: Yes: distribution of savings contingent on quality improvements achieved	factors (age, comorbidities, clinical severity markers). Individual stop loss provision. Exclusions: level 4 neonatal intensive care.	[40]
**9**	New York State's Medicaid Maternity Care Value Based Payment Arrangement (New York States)	US (New York state)	2016	Pilot	Year 1: shared savings (one-sided). Year 2: shared savings (two-sided).	Multiple organisations representing different levels of care	Value Based Payment contractor (usually a hospital and/or professionals involved in maternity care)	All-risk pregnancies	270 days before delivery till 60 days postpartum	Newborn. Prenatal services including visits, lab tests, medication, ultrasounds. Services associated with delivery whether vaginal or caesarean, up to 60 days postpartum for the mother. Facility costs, professional services and associated complications for mother and child are included. Newborn care up to 30 days postpartum.	Year 1: Yes: distribution of savings contingent on achievement of specified quality improvements. Year 2: Yes: distribution of savings contingent on achievement of specified quality improvements, with penalties	Exclusions of mothers aged <12 or > 64 at the time of the delivery, maternal death, stillborn and multiple live births, medicaid members for whom medicaid is not the sole payer, members eligible for inclusion in another program (e.g.	[41-43]

No.	Initiative (abbreviation)	General characteristics			Type of APM							Risk mitigation strategy	Ref
		Country	Year of implementation	Status	Type of APM	Care providers participating in the payment model	Accountable entity	Eligible population	Episode time span	Care activities covered by payment model	Linkage to quality		
											partially or fully written off if quality improvements achieved	hiv/aids or intellectually or developmentally disabled). Risk adjustment and stop loss provision for deciding on the shared savings. Bundles in three episodes: pregnancy, delivery till 60 days post discharge and neonatal care)	
**10**	Pacific Business Group on Health, Blended Case Rate (Pacific)	US (Southern California)	2014	Pilot	Bundled payment light. Fee for service (one blended fee per pregnant woman for all hospital deliveries irrespective of type).	Single health care organisation	Hospital is accountable for the facility blended rate. Medical group practice is accountable for the professional blended rate.	All risk hospital deliveries.	Hospital labour and delivery period only	All facility and professional activities during labor and delivery for both vaginal and caesarean section births.	n.a.	Exclusions of pregancies that left against medical advice, transferred during labor, various comorbidities (e.g. hiv/aids, cancer, also gestational age <37 weeks, multi gestation 3+).No prospective risk	[19, 35, 44-46]

No.	Initiative (abbreviation)	General characteristics			Type of APM							Risk mitigation strategy	Ref
		**Country**	**Year of implementation**	**Status**	**Type of APM**	**Care providers participating in the payment model**	**Accountable entity**	**Eligible population**	**Episode time span**	**Care activities covered by payment model**	**Linkage to quality**		
												adjustment.	
**11**	Minnesota Blended Payment (Minnesota BP)	US (Minnesota)	2009	Permanent implementation	Bundled payment light. Fee for service (one blended fee per pregnant woman for all hospital deliveries irrespective of type).	hospital	Hospital	Uncomplicated births	Delivery	professional services and facility fees for vaginal or casarean delivery and prenatal, postnatal care	no	Exclusion of complicated vaginal deliveries	24, 47
**12**	Minnesota Birth Centers, BirthBundle (BirthBundle)	US (Minnesota)	2015	Pilot stopped	Bundled payment (retrospective). Benchmark based on historical data. If all care is within the birth center, facility fees and professional fees are included in the bundle. No birth center coordination fee in event of hospital delivery. Professional fees only are included if delivered in a hospital.	Single health care organisation	Minnesota Birth Center	Low-risk pregnancies / medium / high -risk	270 days before delivery to 56 days postpartum	Mother and newborn and facility fees. Prenatal care including ultrasounds, lab testing. Perinatal including doula. Postpartum neonatal assessment within 24-hours postpartum and consultation at 1-2 weeks and 6 weeks and lactation support.	n.a.	Facility fees are FFS outside of bundel.	19, 48
**13**	Bundled payment for maternity care (The Dutch BP)	The Netherlands	2017	Pilot	Bundled payment (prospective)	Multiple organisations representing different levels of care	Integrated care organization	All risk pregnancies.	From pregnancy identification to 6 weeks post partum.	Bundle defined in four phases (prenatal, natal, postnatal and kraamzorg), covering all necessary care - according to the national care standardization guidelines.	no (although possible to define links in contract)	Exclusions of bundle breakers (i.e. when pregnant woman decides to see care professionals outside of the integrated	12, 49-51

No.	Initiative (abbreviation)	General characteristics			Type of APM							Risk mitigation strategy	Ref
		Country	Year of implementation	Status	Type of APM	Care providers participating in the payment model	Accountable entity	Eligible population	Episode time span	Care activities covered by payment model	Linkage to quality		
												care network. Depending on the contract, risk corridors on where new negotiations are started - are negotiated. Each phase (except for kraamzorg) destinguishes between regular and complex cases.	
**14**	Maternity Pathway Bundled Payment (Maternity Pathway BP)	England	2013	Permanent implementation	Bundled payment (prospective)	Multiple organisations representing different levels of care	Single lead provider per phase.	Low-, intermediate - and high-risk pregnancies	From 10th week of pregnancy to 6 weeks postpartum	All maternal and neonatal care in the prenatal, perinatal and postpartum phase.	n.a.	Exclusions, stratifying risks in several bundle prices. Supplementary payments for specific complications. in three components: prenatal, perinatal and postpartum. Prenatal and postpartum further stratified into standard, intermediate and intensive categories. Perinatal	52

No.	Initiative (abbreviation)	General characteristics			Type of APM							Risk mitigation strategy	Ref
		Country	Year of implementation	Status	Type of APM	Care providers participating in the payment model	Accountable entity	Eligible population	Episode time span	Care activities covered by payment model	Linkage to quality		
												further stratified into with complications and co-morbidities and without complications and comorbidities. There are supplementary payments for specific complications. Exclusions: health problemes in neonates	
**15**	Providence Health and Services, Pregnancy Care Package (Providence)	US (Oregon)	2013	Permanent implementation	Bundled payment (prospective)	Single health care organisation	Nurse or midwife	Low-risk pregnancies	From pregnancy identification to 6 weeks postpartum	Mother and newborn. All prenatal care, including check-ups, prenatal screening, education, psychosocial support. All delivery-related care and all postpartum care for mother and neonate. Also includes mobile app (Doula) and patient navigators.	n.a.		[19]
**16**	Geisinger Health System, Perinatal ProvenCare Initiative (GHS)	US (Pennsylvania)	2007	Permanent implementation	Bundled payment (prospective)	Multiple organisations representing different levels of care	GHS provider	Low-risk pregnancies.	From pregnancy identification to a consultation 21–56 days	Mother only. Prenatal care including professional and outpatient services. Postpartum care including inpatient readmissions,	No link, although quality metrics are incorporated, measured and tracked.	Exclusions: late referrals, high risk pregnancies, members not enrolled during the	[19, 21, 35]

No.	Initiative (abbreviation)	General characteristics			Type of APM							Risk mitigation strategy	Ref
		Country	Year of implementation	Status	Type of APM	Care providers participating in the payment model	Accountable entity	Eligible population	Episode time span	Care activities covered by payment model	Linkage to quality		
									postpartum	outpatient and professional care.		entire pregnancy (i.e. at least 12 continuous weeks of prenatal care and delivery must be performed by a GHS provider). Exclusions: neonatal care, care delivered by non-GHS providers.	
**17**	Lead Maternity Care Model (LMC)	New Zealand	2007	Permanent implementation	Bundled payment (prospective)	Single provider if the lead maternity caregiver was the actual practitioner, otherwise multiple providers	LMC midwife, GP or obstetrician	All risk pregnancies	From pregnancy identification to 6 weeks postpartum	Mother only. Including complications during postpartum period, including episode-related maternal readmissions,postpartum minimum of 5 home visits till 6 weeks postpartum. Exclusions: neonatal care, ultrasounds, consukIting obstetricians and pediatricians	n.a.	Exclusions of pregnancies that went elsewhere for further care. And exclusions of neonatal care. (fixed budget) in four modules: first and second trimester, third trimester, labour and birth and postnatal care.	53-55
