# Supplementary material for: A Scoping Review of Alternative Payment Models in Maternity Care: Insights in Key Design Elements and Effects on Health and Spending
Source: Int J Integr Care. 2021 Apr 21;21(2):6. doi: 10.5334/ijic.5535 (PMC8086739; doi:10.5334/ijic.5535)
Supplement: Appendix S3. — Full-text document types. [file ijic-21-2-5535-s3.pdf]

Appendix S3 – Full-text document types

Author, year	Document type	Initiative names
Miller et al., 2013	Interviews	LMC
Jarlenski et al., 2016	Commentary	Tennessee, Arkansas and Ohio
Kozhimannil et al., 2018	Evaluation	Minnesota Blended Payment
Carroll et al 2018	Evaluation	Arkansas
Butcher 2018	Blog	Horizon
Dahlen et al 2017	Evaluation	Texas Medicaid Program
HCPLAN	Review	Ohio, Minnesota birth bundle, baby company, AABC (proposal), Pacific, GHS, Providence, CHC, Arkansas, Tennessee
Calvin & Balazovic	White paper	Minnesota Birth Bundle
HCPLAN (experiences from ohio and tennessee)	Review - case report	Ohio and Tennessee
Fish 2017	Powerpoint Presentation	New York DSRIP
RIVM Factsheet	Factsheet	Dutch maternity care bundle
Smith and Hanlon 2017	Case Study	Tennessee
Berry et al., 2011	Observational study	GHS
Henderson 2016	White paper	UK
Ertok, 2015	Evaluation	CQUIN
Lally 2013	Issue brief	PBGH, Arkansas and GHS
New York State 2017	Factsheet	New York DSRIP
Ohio episode based payment update 2014	Powerpoint Presentation	Ohio
Sutherland 2014	Commentary	Ohio
Chernew et al	Interviews	Arkansas
Arkansas Health Care Payment Improvement Initiative. Perinatal episode of care 2017	Program overview	Arkansas
State of Ohio. Detailed business requirements: perinatal episode. 2017	Perinatal Episode description	Ohio
DeRoche et al 2015	Commentary	Tennessee
Grigg and Tracy 2013	Description of NZ's maternity care system	LMC
Tennessee Detailed Business Requirement	Perinatal Episode description	Tennessee
De Brantes and Love 2016 NEJM Catelyst	Catalyst article	CMC Texas
Rubinstein 2015	Case study	Pacific business group,

HCIII Arkansas Episode Design Summary	Perinatal Episode description	Arkansas
De Brantes and Love Case Study		Texas Medicaid
Castellucci et al 2018	Blog	Humana
NZ Ministry 2007	Legal document	LMC
New York State 2016	White Paper	New York DSRIP
Perinatal Practices 2010	Legislative paper	Minnesota Blended Payment
New Zealand Gazette 2018	Legislative paper	LMC
Healthcare Payment Improvement Initiative	White paper	Arkansas
Integrated Healthcare Association 2016	Episode description	Pacific Business Group
