# Supplementary material for: A Scoping Review of Alternative Payment Models in Maternity Care: Insights in Key Design Elements and Effects on Health and Spending
Source: Int J Integr Care. 2021 Apr 21;21(2):6. doi: 10.5334/ijic.5535 (PMC8086739; doi:10.5334/ijic.5535)
Supplement: Appendix S4. — Quality appraisal of the studies performing an effect evaluation of an APM in maternity care. [file ijic-21-2-5535-s4.pdf]

Appendix S4 – Quality appraisal of the studies performing an effect evaluation of an APM in maternity care

			Score[a]						
**Reference**	**Initiative (abbreviation)**	**Payment model**	**Selection Bias**	**Study Design**	**Confounders**	**Blinding**	**Data Collection**	**Withdrawals and Dropouts**	**Global Rating**
Berry et al 2011 [21]	Geisinger Health System, Perinatal ProvenCare Initiative (GHS)	bundled payment prospective	1	2	3	1	3	n.a.	3
Carroll et al 2018 [23]	Arkansas Health Care Payment Improvement Initiative (Arkansas)	shared savings two sided	1	2	1	1	3	n.a.	2
Kozhimannil 2018 [24]	Minnesota Blended Payment	FFS blended case rate	1	2	1	1	3	n.a.	2
Dahlen et al 2017 [22]	Texas Medicaid Program	FFS with penalties	1	2	1	1	3	n.a.	2

a Scores of 1, 2, and 3 correspond to strong, moderate, and weak quality, respectively, using the Effective Public Health Practice Project Quality Assessment Tool for Quantitative Studies 22 .
